# Screening Probiotics for Anti-*Helicobacter pylori* and Investigating the Effect of Probiotics on Patients with *Helicobacter pylori* Infection

**DOI:** 10.3390/foods13121851

**Published:** 2024-06-13

**Authors:** Hui Yang, Yang Lin, Yuchan Ma, Jiaru Li, Junxiang Li, Zeqi Huo, Pingrong Yang, Chunjiang Zhang

**Affiliations:** 1 School of Pharmacy, Lanzhou University, Lanzhou 730000, China; 2 School of Life Sciences, Lanzhou University, Lanzhou 730000, China; 3 Key Laboratory of Cell Activities and Stress Adaptations, Ministry of Education, Lanzhou University, Lanzhou 730000, China; 4 Gansu Key Laboratory of Biomonitoring and Bioremediation for Environmental Pollution, Lanzhou University, Lanzhou 730000, China

**Keywords:** *Helicobacter pylori*, *Lactobacillus plantarum*, inflammatory, gut microbiota, metabolomics

## Abstract

Probiotics are natural microbial agents with beneficial properties such as bacteriostatic and anti-infective properties. *Lactobacillus plantarum* Q21, Q25 and QA85, were isolated from the Chinese specialty fermented food “Jiangshui” and proved to be highly resistant to *Helicobacter pylori* (*p* < 0.0001). In vitro results showed that Q21, Q25 and QA85 strongly inhibited *H. pylori* and could specifically co-aggregate *H. pylori* in vitro (more than 56%). Strains have the potential to adhere to cells and hinder *H. pylori* colonization (*p* < 0.0001). To assess the anti-*H. pylori* efficacy of strains in vivo, volunteers were recruited and a self-controlled study of probiotic intervention was conducted. Compared to pre-probiotics, volunteers who took Q21, Q25 and QA85 for 1 month showed significant improvement in discomfort, a significant reduction in GSRS scores (*p* < 0.05), and modulation of inflammatory response (*p* < 0.05). Q21, Q25 and QA85 resulted in a decreasing trend of *H. pylori* load in volunteers (454.30 ± 327.00 vs. 328.35 ± 237.19, *p* = 0.06). However, the strains were not significantly effective in modulating the imbalance of the gut microbiota caused by *H. pylori* infection. In addition, strains affect metabolic pathways by increasing the levels of O-Phosphoethanolamine and other related metabolites, which may ameliorate associated symptoms. Therefore, *Lactobacillus plantarum* Q21, Q25 and QA85 can be regarded as a candidate probiotic preparation that exerts direct or indirect anti-*H. pylori* effects by inhibiting *H. pylori* activity and colonization, reducing inflammation and discomfort, maintaining homeostasis in the internal environment, affecting the metabolic pathways and repairing the body barrier. They can play a role in relieving *H. pylori* infection.

## 1. Introduction

As a common human pathogen, *Helicobacter pylori* infects over 50% of the global population [1]. In some regions of Latin America, Africa, and Asia, the adult infection rate even exceeds 75% [2,3]. Despite lower infection rates in children, it still reaches as high as 32.3% globally, showing a significant negative correlation with income levels among countries or regions [4]. Given the potential health risks associated with *H. pylori* infection, including chronic progressive gastritis, peptic ulcer disease, gastric atrophy, and gastric epithelial dysplasia, as well as the eventual progression to mucosa-associated lymphoid tissue lymphoma or gastric cancer [5], timely detection and intervention for *H. pylori* are necessary. Currently, standard triple or quadruple therapy is commonly employed clinically for *H. pylori* infection, which includes two antibiotics and a proton pump inhibitor (PPI), with quadruple therapy adding bismuth salts to this regimen. Although the above methods are effective in eradicating *H. pylori*, they still face many challenges and shortcomings, such as the problem of antibiotic resistance rate [6], the safety of bismuth agents [7], the limitations of some of the PPI formulations, and patient compliance [8], among others. Therefore, there is still a need for further expansion of treatment methods for *H. pylori* infection.

Probiotics are living microorganisms that can play a beneficial role in the host’s health by ingesting a certain amount [9], primarily including lactobacilli, bifidobacteria, certain streptococci and yeasts [10]. Probiotics have the ability to inhibit the growth of pathogenic bacteria, regulate the microecological balance within the body, and treat diseases caused by pathogenic bacteria, such as acute diarrhea infections and colitis [11]. Additionally, probiotics contribute to enhanced digestion and absorption, as well as the regulation of the immune system, all without adverse effects [12]. Currently, a variety of probiotic preparations have been clinically applied in the treatment of diseases caused by gastrointestinal pathogenic bacterial infections [13,14]. Several studies have demonstrated the effectiveness of various probiotics in the treatment of diseases resulting from *H. pylori* infections, such as *L. acidophilus* [15,16], *L. rhamnosus* GG [17], *L. casei* [18,19], *L. gasseri* OLL2716 [20], *L. reuteri* [21], *L. plantarum* [22,23], *Bifidobacterium* [24,25], and others. Previous studies have shown that probiotics inhibit *H. pylori* by producing substances that inhibit *H. pylori* urease [26,27], blocking the adhesion of *H. pylori* to gastrointestinal epithelial cells, and inhibiting the inflammatory response after *H. pylori* infection [28], among other mechanisms.

Probiotic supplementation in the treatment of *H. pylori* may increase eradication rates and may reduce the incidence of adverse effects [29,30]. However, there is still no definitive conclusion on whether probiotics can reduce the load of bacteria. Some studies have shown that taking probiotics can reduce the bacterial load of *H. pylori* and lessen the severity of gastritis [31]. Probiotics are also used to treat diseases that can lead to disturbances in the gut microbiota [32]. However, the regulatory effects of probiotics on the gut microbiota [33] and their influence on fecal metabolites remain unclear.

One of the main sources of isolated *Lactobacillus* is fermented foods, and Jiangshui is one of the specialty fermented foods in Northwest China, which is very popular among the public. Additionally, some studies have found that *Lactobacillus* is the dominant species in the microbial composition of Jiangshui [34]. Thus, Jiangshui can be used as an important source for isolation of *Lactobacillus*. Early studies related to probiotics (e.g., *Bifidobacterium*, *L. acidophilus*, and *L. casei*) on *H. pylori* focused on the inhibition of *H. pylori* by probiotics [15,18,19,24], or the effect of probiotics combined with standard therapy on patient eradication rates. Therefore, in this study, we proposed to screen several strains of *Lactobacillus plantarum* isolated from Jiangshui, a specialty fermented food in Northwest China, with strong anti-*H. pylori* ability. While focusing on the inhibitory effect of probiotics on *H. pylori*, the efficacy of probiotics in alleviating the symptoms associated with *H. pylori* infection in volunteers by administration of probiotics alone was explored, with attention to their role in influencing the gut microbiota and fecal metabolites.

## 2. Materials and Methods

### 2.1. Bacterial Strains and Culture Conditions

From more than 100 strains independently isolated in the laboratory, seven strains with strong bacterial inhibitory ability were screened: ZCJ, TSL-6, QX(A)-4, GL-5, Q21, QA85, and Q25. These seven strains were identified as *Lactobacillus plantarum* by sequencing, and the conservation information is shown in Table S1. Seven strains and one control strain *Lactobacillus rhamnosus* GG (LGG) were stored at −80 °C in MRS broth containing 20% glycerol (*v*/*v*) until tested. LGG is a widely used commercial probiotic strain and hence was used in this study. LGG was stored in a lab at Lanzhou University (China). The strains were cultured in MRS broth at 37 °C for 24 h to activate *Lactobacillus*, repeated twice and then subjected to each experiment. *H. pylori* J99 was stored in the Lanzhou University Microbiological Culture Collection and stored at −80 °C in *H. Pylori* Medium (fluids) containing 20% glycerol (*v*/*v*) until tested. *H. pylori* was grown on Columbia blood agar plates containing 5% (*v*/*v*) off fiber sheep blood (Qingdao Hope Bio-Technology Co., Ltd., Qingdao, China) at 37 °C in microaerophilic conditions (5% O_2_, 10% CO_2_, and 85% N_2_).

### 2.2. Cell Culture

AGS cells (human gastric adenocarcinoma epithelial cells, ATCC CRL 1739) were purchased from Procell Life Science and Technology Co., Ltd. (Wuhan, China). AGS cells were maintained in RPMI 1640 medium supplemented with 15% fetal bovine serum (FBS) (Gemini Fetal Bovine Serum, Gemini Biotechnology Company, West Sacramento, CA, USA), penicillin (100 IU/mL), and streptomycin (100 μg/mL) at 37 °C with 5% CO_2_.

### 2.3. Screening for Strains with H. pylori Inhibition Ability

#### 2.3.1. Acid Tolerance of *Lactobacillus*

After activation of *Lactobacillus*, the supernatant was discarded by centrifugation. The bacterial precipitate was washed three times with phosphate-buffered saline (PBS) and resuspended to a viable bacterial count of 10^8^ CFU/mL. *Lactobacillus* suspension with a viable count of 10^8^ CFU/mL was inoculated into simulated gastric juice (0.2% NaCl, 0.35% pepsin, dissolved in distilled water and adjusted to pH 3.0 with HCl; filtered through a 0.22 μm membrane), and sampled at 0 and 3 h of the test, respectively. Counts of viable bacteria were performed using the MRS agar plates method and the survival rate of several *Lactobacillus* strains in simulated gastric juice after 3 h was calculated separately [35].

#### 2.3.2. Hydrophobic Properties of *Lactobacillus*

The hydrophobicity of the bacterial surface was determined according to the method of Kos et al. [36]. After the activation of *Lactobacillus*, the bacteria were washed and resuspended with PBS to give a viable bacterial count of 10^8^ CFU/mL. Then, 3 mL of *Lactobacillus* suspension and 1 mL of xylene were thoroughly mixed, shaken thoroughly for 5 min, allowed to stand at room temperature, and the absorbance values of the aqueous phase were measured after 0 h and 1 h to calculate the hydrophobicity of *Lactobacillus*.

#### 2.3.3. Aggregation Properties of *Lactobacillus*

Autoaggregation properties of *Lactobacillus*: After activation, the *Lactobacillus* was washed and resuspended with PBS, so that the number of viable bacteria was 10^8^ CFU/mL. The mixture was incubated at 37 °C, and the absorbance value at 600 nm of the upper layer liquid was measured within 24 h to calculate the autoaggregation rate of *Lactobacillus* [36,37].

Coaggregation properties of *Lactobacillus* with *H. pylori*: After the activation of *Lactobacillus*, it was washed and resuspended with PBS, so that the number of viable bacteria was 10^8^ CFU/mL. The treatment of *H. pylori* was the same as the operation described above. Then, 5 mL of *Lactobacillus* suspension and 5 mL of *H. pylori* suspension were thoroughly mixed, and we set the mixture at 37 °C for static incubation. Test the absorbance value at 600 nm in the upper layer liquid within 24 h. Calculate the coaggregation rate of the interaction between the *Lactobacillus* and *H. pylori* [36,37].

#### 2.3.4. Anti-*H. pylori* Activity of *Lactobacillus*

Anti-*H. pylori* activity of *Lactobacillus* was determined by the Oxford cup diffusion method. First, 200 μL of *H. pylori* suspension was evenly spread on Columbia blood agar plates without antibiotics. After spreading, 4 sterilized Oxford cups were placed on the plates, and we added 200 μL of *Lactobacillus* suspension, *Lactobacillus* supernatant, amoxicillin solution at a concentration of 0.06 μg/mL (MIC), and a blank MRS broth to Oxford cups, respectively. The plates were incubated at 37 °C under a microaerobic environment for 72 h. At the end of incubation, the diameter of the inhibition circle was measured by vernier caliper [18,38].

#### 2.3.5. Urease Activity Assay

To analyze the inhibitory effects of *Lactobacillus* against *H. pylori* urease, 40 μL of *H. pylori* suspension was mixed with 10 μL of *Lactobacillus* supernatant, and 10 μL of sterile *H. pylori* liquid medium was used as control. The mixture was added to a clean and sterile 96-well plate and incubated at 37 °C in a microaerobic environment for 48 h. The incubated mixture was taken out, and 150 μL of urease reagent (20% urea, 0.012% phenol red, dissolved in PBS and adjusted to pH 6.5 with HCl) was added into each well, and then the color change was observed and the OD_550nm_ value was measured [18].

### 2.4. Co-Culture Assay of Lactobacillus with H. pylori

Activated *H. pylori* were resuspended in fresh *H. pylori* liquid medium, and we added 10% *Lactobacillus* suspension (10^8^ CFU/mL) or supernatant. It was co-cultured for a certain period of time and *H. pylori* viable cells were counted using their selective medium. At the same time, urease activity was measured.

### 2.5. Anti-H. pylori Effects of Lactobacillus at the Cell Level

#### 2.5.1. Adhesion Assay of *Lactobacillus* to AGS Cells

To determine the adhesion capacity of *Lactobacillus*, *Lactobacillus* were centrifuged to collect the bacterial bodies and washed with PBS, followed were resuspended with antibiotic-free RPMI 1640 culture medium and adjusting the concentration of *Lactobacillus* to 10^6^, 10^7^, 10^8^, 10^9^, and 10^10^ CFU/mL, respectively, and set aside. Cell spreading was performed by adding 1 mL of 100 × 10^4^/mL cell solution per well in a six-well plate, and the cells were cultured to form a monolayer by adhering to the wall. Subsequently, 1 mL *Lactobacillus* suspension of different concentrations was added to each well, and the experiments were carried out by co-culturing for 4 h at 37 °C. In the end, unadhered bacteria were washed away and the mixture was collected in a centrifuge tube, diluted stepwise and spread on MRS agar plates for counting. The percentage of bacterial adherence to cells was calculated by the following formula: adherence rate (%) = (adherent bacteria)/(total bacteria) × 100. Cell adhesion assay was conducted in biological triplicate to ensure reproducibility.

In order to determine the effect of co-culture duration on adhesion, *Lactobacillus* was adjusted to 10^8^ CFU/mL, and co-cultured with AGS cells (100 × 10^4^ cells) for 1, 2, 4, and 6 h, respectively. Other operations were the same as above.

#### 2.5.2. Inhibition Assay of *H. pylori* Adhesion to AGS Cells by *Lactobacillus*

*Lactobacillus* at a concentration of 10^8^ CFU/mL was resuspended in an antibiotic-free RPMI 1640 culture medium and set aside. *H. pylori* was similarly manipulated. Six-well plates were used for AGS cell spreading (100 × 10^4^ cells), and cells were cultured to form monolayers for experiments. To determine the inhibitory effect of *Lactobacillus* on *H. pylori* adhesion to AGS cells, it was validated in four sets of experiments. Cells were infected with *H. pylori* (MOI = 100) and incubated at 37 °C for 4 h as an infected control. After co-culturing the cells with *H. pylori* (MOI = 100) for 2 h, unadhered bacteria were washed away with PBS, and then *Lactobacillus* (MOI = 100) was added to co-culture with the cells for 2 h as a displacement experimental group. After co-culturing the cells with *Lactobacillus* (MOI = 100) for 2 h, unadhered bacteria were washed away with PBS, and then *H. pylori* (MOI = 100) was added and co-cultured with the cells for 2 h as the exclusion experimental group. The cells were co-cultured with *Lactobacillus* (MOI = 100), *H. pylori* (MOI = 100) for 4 h as a competition experimental group. In the end, unadhered bacteria were washed away and the mixture was collected in a centrifuge tube, and *H. pylori* selective medium was used for counting. The relative percentage of *H. pylori* adhesion to cells was calculated according to the following formula: relative adhesion (%) = (number of adherent bacteria in the experimental group)/(number of adherent bacteria in the control group) × 100. The cell adhesion assay was performed using the biological triplex method to ensure reproducibility.

#### 2.5.3. Total RNA Extraction and Reverse-Transcription Quantitative PCR (RT-qPCR)

To study the anti-inflammatory effect of *Lactobacillus*, it was verified in four groups of experiments. AGS cells (100 × 10^4^ cells) were cultured normally for 4 h as a blank control group. AGS cells were treated with *H. pylori* (MOI = 100) for 4 h as the *H. pylori*-infected group. After co-culturing the cells with *Lactobacillus* (MOI = 100) for 2 h, the unadhered bacteria were washed away with PBS, and then *H. pylori* (MOI = 100) was added and co-cultured with the cells for 2 h as the exclusion experimental group. The cells were co-cultured with *Lactobacillus* (MOI = 100) and *H. pylori* (MOI = 100) for 4 h as the competition experimental group.

Total mRNA was extracted with SevenFast^®^ Total RNA Extraction Kit for Cells (Seven Innovation (Beijing) Biotech Co., Beijing, China). cDNA synthesis was performed with SevenClever ™ First Strand cDNA Synthesis Kit (with dsDNase) (Seven Innovation (Beijing) Biotech Co., Beijing, China). RT-qPCR was performed in qPCR 96-well plates on an Mx3000/Mx3005P system (Agilent, Santa Clara, CA, USA) and 2× SYBR Green qPCR MasterMix II (Universal) (Seven Innovation (Beijing) Biotech Co., Beijing, China) to analyze the TNF-α, IL-6, IL-8 and IL-10 mRNA expressions. The oligonucleotide sequence of primers used for RT-qPCR [39,40,41] is listed in Table 1.

#### 2.5.4. Cytotoxicity Assay on AGS Cells

The cytotoxicity effect of *Lactobacillus* on AGS cells was determined by MTT assay. Firstly, AGS cells (10^4^ cells) were grown and allowed to adhere to a 96-well plate at 37 °C for 12–24 h to approximately 80% confluence and then cells were treated at 37 °C in a 5% CO_2_ atmosphere with *Lactobacillus* (MOI = 100) for 6 h. Then 20 μL of MTT solution (5 mg/mL) was added to each well, and the cells were incubated for 4 h at 37 °C. Finally, 150 μL of DMSO was added to each well and we allowed the color to develop. The absorbance was measured at 490 nm. Cell viability (%) = (Asample/Acontrol) × 100, where Asample is the absorbance of the cells that were incubated with the medium containing *Lactobacillus*, and Acontrol is the absorbance of the cells alone.

### 2.6. Antibiotic Susceptibility Assay

The susceptibility of *Lactobacillus* to antibiotics was determined by the disc diffusion method [42]. The concentration of *Lactobacillus* was adjusted to 10^8^ CFU/mL, and the bacterial suspension was evenly spread on MRS agar plates and left to dry for 5 min. Use tweezers to stick the antibiotic discs (Microbial Reagent Co., Ltd., Hangzhou, China) onto the surface of the inoculated plates, and incubate in an inverted at 37 °C for 24 h, with the quality control bacteria as a control. The diameter of the inhibition circle was measured and recorded by vernier caliper. Three replicates were set up for each antibiotic and the results were averaged. The standard strains of *Staphylococcus aureus* ATCC 25923 (*S. aureus*) and *Escherichia coli* ATCC 25922 (*E. coli*) were used as the control bacteria, operating as above. The inhibition zone after incubation was measured and interpreted as susceptible, intermediate, or resistant according to the instructions for the antibiotic discs (Microbial Reagent Co., Ltd., Hangzhou, China).

### 2.7. Effects of Lactobacillus on Patients with H. pylori Infection

#### 2.7.1. Participating Volunteers

Volunteer selection such as inclusion and exclusion criteria were referred to the experimental design of Chenghai Yang [43]. Recruited volunteers should be tested with a ^14^C urea breath test (^14^C-UBT) before starting the trial and should be included in the trial if they are positive for *H. pylori*. A total of 94 volunteers were recruited for the trial, among whom 49 tested positive for *H. pylori* by ^14^C-UBT and were eligible for the trial. Volunteers underwent a one-month intervention study using probiotics. In total, 37 volunteers participated in the entirety of the trial.

This study was reviewed by the Ethics Committee of the School of Life Sciences, Lanzhou University, China (No. EAF2023052, 26/9/2023) and adhered to the Declaration of Helsinki. All volunteers were fully aware of the possible benefits and potential risks of participating in the trial and voluntarily signed a written informed consent form.

#### 2.7.2. Study Design

This intervention study was designed as a self-control test before and after taking probiotics volunteers. The dietary intervention used probiotic preparations, and the intervention study lasted for 1 month. The volunteers before and after taking probiotics were named as M0 and M1 groups, respectively. The study used probiotic powder (solid drink) as a way to give probiotics to volunteers. Volunteers were given probiotic powder (1 × 10^11^ CFU/packet) with warm water, twice a day, one packet each time before breakfast and dinner, for a total of one month. The probiotic product contained 1 × 10^11^ CFU of Q21, Q25 and QA85 in each packet, prepared as a solid powder (WECARE-BIO Biological Technology Co., Ltd., Suzhou, China) for oral application.

#### 2.7.3. Volunteer Basic Information Statistics and ^14^C-UBT Detection

Before and after the test, volunteers were tested for ^14^C-UBT. This non-invasive method is frequently used in the clinical diagnosis of *H. pylori* infection, and the sensitivity and specificity of this test usually exceeds 95% [44]. A ^14^C-UBT detection value ≥ 100 is clinically diagnosed as positive for *H. pylori* infection, and using this diagnostic principle, volunteers were registered for the study. Basic information such as demographic data and medical history was also collected.

#### 2.7.4. Gastrointestinal Symptom Assessment

Referring to the questionnaire designed by Svedlund et al. [45,46], volunteers were asked to recall the history of gastrointestinal symptoms at the beginning of the trial, to observe changes in symptoms during the treatment period, and to fill in the Gastrointestinal Symptom Rating Scale (GSRS) after 1 month. The scale’s symptom scores from mild to severe corresponded to a score of 1–7, and the level of the score was directly proportional to the severity of the gastrointestinal symptoms. Gastrointestinal symptoms were assessed according to the GSRS before (M0) and after the probiotics diet (M1). Pay special attention to symptoms such as acid regurgitation, nausea, abdominal distension, diarrhea, and constipation.

#### 2.7.5. Measurement of Cytokines

We collected serum from volunteers before and after taking probiotics. The levels of inflammatory cytokines (TNF-α, IFN-γ, IL-1β, IL-6, IL-8, IL-10, IL-17) in serum were analyzed by ELISA (Shanghai Kexing Trading Co., Ltd., Shanghai, China) kits according to the instructions.

#### 2.7.6. Analysis of Gut Microbiota

Fecal samples were collected from volunteers before and after the administration of probiotics. Volunteers were instructed to collect their fecal samples at the testing sites using sterile disposable fecal collection tubes. Samples were promptly stored at −80 °C upon collection. The fecal samples were sent to Suzhou PANOMIX Biomedical Tech Co., Ltd., Suzhou, China for 16S rRNA gene sequencing. Genomic DNA was extracted from fecal samples using the DNA Strong Extraction Kit (Omega, Tarzana, CA, USA), following the instructions. DNA was also quantified by Nanodrop (Thermo Fisher Scientific, Waltham, MA, USA) and the quality of DNA extraction was checked by 1.2% agarose gel electrophoresis. According to the concentration, DNA was diluted to 20 ng/μL using sterile water. PCR primers containing a barcode (338F 5′-ACTCCTACGGGAGGCAGCA-3′, 806R 5′-GGACTACHVGGGTWTCTAAT-3′) were used to amplify the bacterial 16S rRNA variable region V3–V4. Amplification conditions: initial denaturation 98 °C 2 min; denaturation 98 °C 15 s; annealing 55 °C 30 s; extension 72 °C 30 s; final extension 72 °C 5 min; 10 °C holding; 25–30 cycles. Subsequently, gel excision was performed targeting the desired bands to obtain purified samples (AxyPrep DNA Gel Extraction Kit, Axygen, Corning, NY, USA). Individual samples were also quantified using a BioTek enzyme labeler (BioTek Flx800 enzyme labeler; Quant-iT PicoGreen dsDNA Assay Kit, Invitrogen, Waltham, MA, USA). Finally, the standard Illumina TruSeq DNA library preparation procedure (Illumina TruSeq DNA Sample Preparation Guide) was used to construct the required onboard libraries. After the libraries passed the test, they were sequenced using NovaSeq6000. Sequencing and data processing were performed by Suzhou PANOMIX Biomedical Tech Co., Ltd., Suzhou, China.

#### 2.7.7. Metabolomics Testing and Analysis

The fecal sample was weighed accurately in a 2 mL centrifuge tube, and 600 µL of methanol containing 2-chloro-L-phenylalanine (4 ppm) was added and vortexed for 30 s. The samples with steel beads were added to a tissue grinder and ground at 50 Hz for 120 s. The samples were ultrasonicated for 10 min at room temperature and centrifuged at 12,000 rpm, 4 °C for 10 min. The supernatant was filtered through a 0.22 μm membrane and the filtrate was added to the detection vial for LC-MS detection. Sequencing and data processing were performed by Suzhou PANOMIX Biomedical Tech Co., Ltd., Suzhou, China.

### 2.8. Statistical Analysis

The data were expressed as the mean ± standard deviation (SD). All the experiments were conducted at least in triplicate. Differences were determined by One-way analysis of variance (ANOVA) and Dunnett’s test, using GraphPad Prism 9 (GraphPad Software, Inc., San Diego, CA, USA). Differences between the two groups were determined by Independent-Samples T-Test, using IBM SPSS Statistics 26 (IBM SPSS Inc., Chicago, IL, USA). A *p*-value less than 0.05 was considered as a significant difference.

## 3. Results

### 3.1. Three Strains of Lactobacillus with Strongly Inhibitory Effect on H. pylori

The results showed that eight strains could survive for 3 h under artificial gastric juice at pH 3.0, with four strains surviving more than 20% (Figure S1A). The hydrophobicity of these eight strains of *Lactobacillus* with acid resistance was analyzed (Figure S1B). The results showed that the hydrophobicity of the *Lactobacillus* ranged from 12–31%, and QA85 was the most hydrophobic.

The aggregation of bacterial strains is divided into two forms: autoaggregation and coaggregation. From the results, most of the *Lactobacillus* showed strong autoaggregation ability, and the autoaggregation increased with the increase in incubation time. When the incubation time was 24 h, Q25 showed the strongest autoaggregation at 69%, while autoaggregation rates of QA85, Q21, and LGG were also high (above 60%) (Figure S1C). Coaggregation results showed that *Lactobacillus* and *H. pylori* coaggregation also increased with time. The maximum coaggregation rate of QA85 was 60% at an incubation time of 24 h (Figure S1D). The aggregation of pathogenic bacteria by *Lactobacillus* can make it easier for pathogenic bacteria to be eliminated from the intestine.

**Figure 1:**
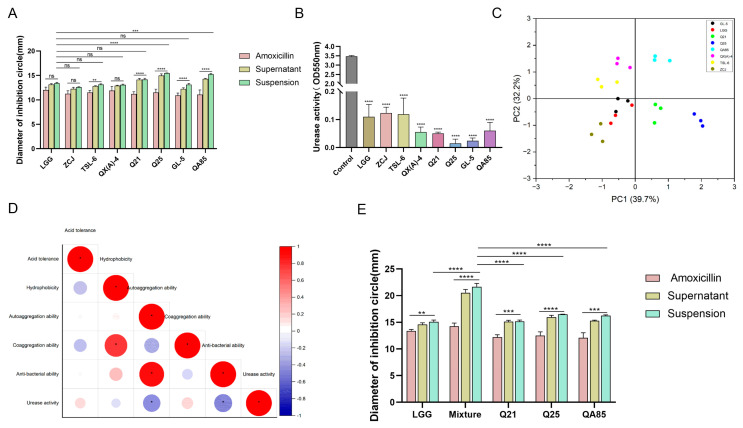
Screening *Lactobacillus* and testing the effect of the mixture. (**A**) Determination of the inhibitory ability of *Lactobacillus* against *H. pylori*. (**B**) Determination of urease activity (OD_550nm_). (**C**) Factor score plots of influencing factors. (**D**) Correlation analysis of influencing factors. (**E**) Determination of the inhibitory capacity of a mixture of Q21, Q25, and QA85 against *H. pylori*. Error bars represent the standard deviation of biological triplicates. *, *p* < 0.05; **, *p* < 0.01; ***, *p* < 0.001; ****, *p* < 0.0001; ns, no significant difference.

From the bacterial inhibition assay, it can be seen that the amoxicillin inhibited the growth of *H. pylori* and the circle of inhibition was around 10–12 mm (Figure 1A). The inhibition circle of *Lactobacillus* suspensions was in the range of 12–16 mm, and the inhibition circle of *Lactobacillus* supernatants was in the range of 12–15 mm. The strongest ability to inhibit *H. pylori* was Q25. The expression level and activity of urease are critical for the survival of *H. pylori* in an acidic environment. It was demonstrated that the supernatants of *Lactobacillus* significantly inhibited the urease activity of *H. pylori*, with Q25 having the strongest inhibitory ability (Figure 1B).

It was found through the factor scoring plot (Figure 1C) that the first component accounted for 39.7% and the second component accounted for 32.2%, with the bacteriostatic ability playing a major contributing role. From the correlation analysis (Figure 1D), there was a close association between the nature of *Lactobacillus* and its inhibition of *H. pylori* growth. Therefore, based on the principal component analysis and the properties of *H. pylori* growth inhibition, Q21, Q25, and QA85 were screened as potential anti-*H. pylori* probiotics and were used in subsequent studies. The three strains were compounded to verify the antibacterial effect of the mixture (1:1:1). It was found that the mixture also possessed better antibacterial ability (*p* < 0.0001) (Figure 1E).

### 3.2. Co-Culture Validation Results of Lactobacillus and H. pylori

**Figure 2:**
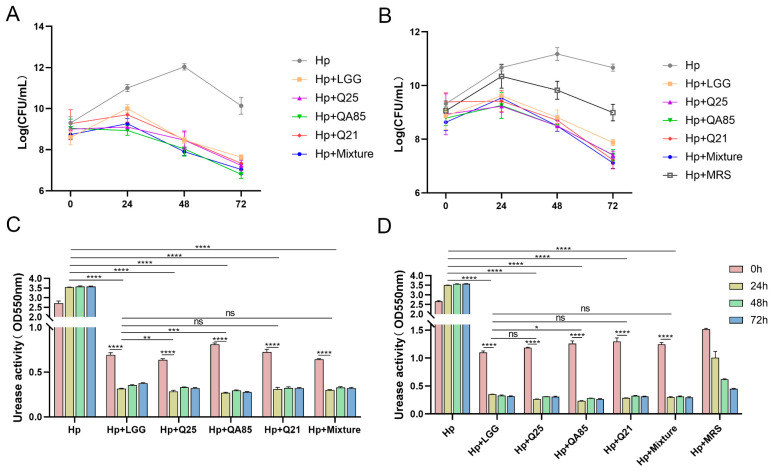
Co-culture validation experiments of *Lactobacillus* and *H. pylori*. Counting of live *H. pylori* in co-culture of *Lactobacillus* suspension (**A**) and supernatant (**B**) with *H. pylori*. Measurement of urease activity in co-culture of *Lactobacillus* suspension (**C**) and supernatant (**D**) with *H. pylori*. Error bars represent the standard deviation of biological triplicates. *, *p* < 0.05; **, *p* < 0.01; ***, *p* < 0.001; ****, *p* < 0.0001; ns, no significant difference.

Q21, Q25, and QA85 were screened as potential strains of anti-*H. pylori*. To further confirm the ability of *Lactobacillus* to antagonize *H. pylori*, with LGG as a commercial control strain, we analyzed the effect of *Lactobacillus* suspension and supernatant on the inhibition of *H. pylori* growth under co-culture conditions. From the effect of *Lactobacillus* on the growth and urease activity of *H. pylori* (Figure 2), the number of viable bacteria of *H. pylori* decreased with the extension of time. At 24 h, all three strains inhibited *H. pylori* growth and urease activity, with Q25 being the most potent strain in inhibiting *H. pylori*. At 48 h and 72 h, the strains also inhibited *H. pylori* growth and urease activity.

### 3.3. Adhesion of Lactobacillus to AGS Cells and Inhibition of H. pylori Adhesion

**Figure 3:**
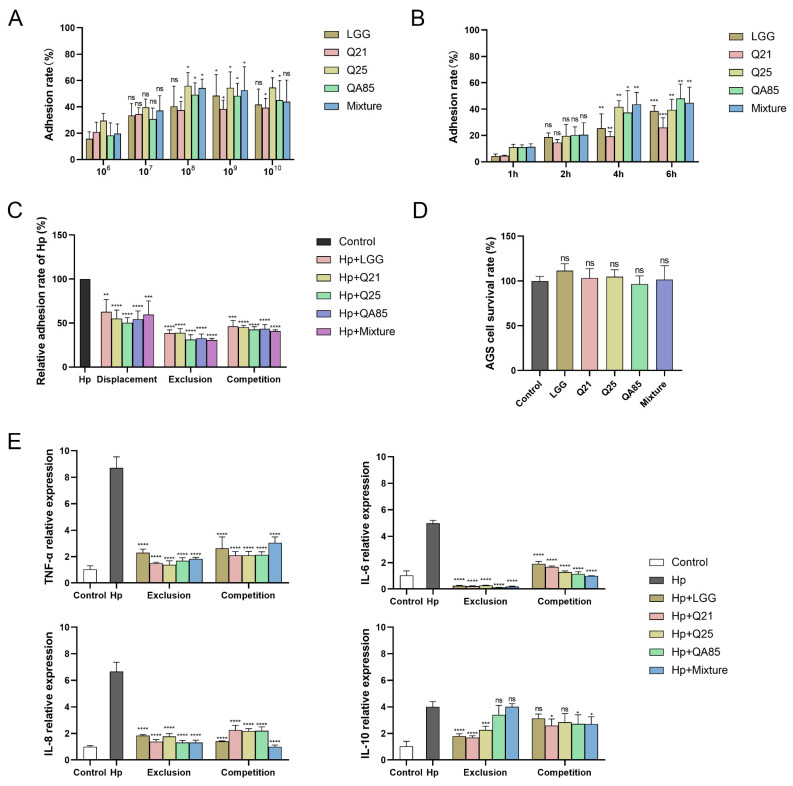
Functional validation of probiotics at the cell level. (**A**) Adhesion at different concentrations of *Lactobacillus* with AGS cells (10^6^ cells). (**B**) Adhesion of *Lactobacillus* (10^8^ CFU/mL) to AGS cells (10^6^ cells) at different time durations. (**C**) Inhibition of *H. pylori* (MOI = 100) adhesion to AGS cells by *Lactobacillus* (MOI = 100). (**D**) Cell viability was determined by MTT assay after 6 h of co-culture (MOI = 100). (**E**) TNF-α, IL-6, IL-8 and IL-10 expression levels in AGS cells treated with *H. pylori* and different *Lactobacillus*. Error bars represent the standard deviation of biological triplicates. *, *p* < 0.05; **, *p* < 0.01; ***, *p* < 0.001; ****, *p* < 0.0001; ns, no significant difference.

To confirm the ability of several strains to adhere to gastric epithelial cells, their adhesion rates to AGS cells were explored. Firstly, the adhesion rate of *Lactobacillus* on AGS cells at different concentrations was explored (Figure 3A), and it was found that the adhesion rate of *Lactobacillus* at a concentration of 10^8^ CFU/mL was significantly different compared with 10^6^ CFU/mL (*p* < 0.05). Additionally, the adhesion rate of *Lactobacillus* on AGS cells was explored at different incubation times (Figure 3B), and there was a significant difference in the adhesion rate at 4 h or 6 h compared with 1 h of incubation (*p* < 0.01, *p* < 0.001).

Since the screened strains had a strong ability to adhere to AGS cells, we established three cellular models, displacement, exclusion and competition, to explore the potential of the strains to hinder *H. pylori* colonization. The results showed that these strains inhibited *H. pylori* adhesion to AGS cells (Figure 3C). Specifically, the relative adhesion rates of *H. pylori* containing Q21, Q25, QA85, and their mixture were 55.04%, 50.39%, 54.26%, and 59.69%, respectively, compared with *H. pylori* adhesion to AGS cells alone during displacement adhesion group (*p* < 0.0001, *p* < 0.001). Their relative adhesion rates were 38.76%, 31.16%, 32.79%, and 30.85%, respectively, during the exclusion adhesion group (*p* < 0.0001), and the mixture inhibited adhesion at the highest rate. Their relative adhesion rates were 45.12%, 42.56%, 43.49%, and 40.85% (*p* < 0.0001), respectively, during the competition adhesion group, and the mixture still had the highest inhibition rate of adhesion. Taken together, the relative adhesion rate of *H. pylori* in the exclusion group was lower than the other two modes, indicating that the strains had a high ability to hinder *H. pylori* adhesion under the exclusion conditions, with an inhibition adhesion rate of more than 61%, and the inhibition rate of the mixture was the highest. In the competition group, the inhibition adhesion rate of each strain could reach more than 54%, demonstrating the hindrance of strains to *H. pylori*.

*H. pylori* infection induces the production of inflammatory factors and contributes to inflammation in gastric. The exclusion and competition modes were found to be superior to the displacement mode by *H. pylori* adhesion inhibition with *Lactobacillus*. Therefore, these two modes were chosen for the study of cytokine levels. From the results, the relative expression levels of TNF-α, IL-6, and IL-8 were significantly increased in *H. pylori*-treated AGS cells compared to the control group, and after treatment with Q21, Q25, QA85, and mixture, their relative expression levels were significantly decreased compared to the *H. pylori*-treated group (*p* < 0.0001) (Figure 3E). The results also revealed that compared with the *H. pylori*-infected group, after treatment with *Lactobacillus*, there was a significant change in the relative expression level of IL-10 in the exclusion group and an insignificant change in the competition group. Taken together, the effect of probiotics on the expression of pro-inflammatory factors was much greater than anti-inflammatory factors.

To test the safety of *Lactobacillus* in future applications, we performed the cell viability assay to determine the cytotoxicity of three *Lactobacillus* strains and their mixture to AGS cells (Figure 3D). The results showed that the co-culture of Q21, Q25, and QA85, and their mixture with cells for 6 h had no significant cytotoxic effects on cells.

### 3.4. Antibiotic Susceptibility Analysis of Lactobacillus

Antibiotic susceptibility was an important index for the safety of *Lactobacillus* before application. Therefore, we tested the susceptibility of these *Lactobacillus* to 22 antibiotics (Figure S2). As shown in Table 2, Q21, Q25, QA85, and LGG showed varying degrees of drug sensitivity to 22 antibiotics, with sensitivity to penicillin (e.g., ampicillin), tetracyclines (e.g., doxycycline), cephalosporins (e.g., imipenem), and chloramphenicol; and showed intermediate sensitivity to aminoglycosides and macrolides (e.g., gentamicin and erythromycin). In contrast, these strains were resistant to quinolones (e.g., ciprofloxacin and levofloxacin).

### 3.5. The Effect Analysis of Lactobacillus on Patients with H. pylori Infection

#### 3.5.1. Trial Profile and Volunteer Characteristics

In total, 49 volunteers tested positive for *H. pylori* by ^14^C-UBT and were eligible for the trial. Volunteers before and after taking probiotics were used as M0 and M1 groups of the trial, respectively. Since 12 volunteers dropped the probiotics halfway through the program, a total of 37 volunteers were eligible for the protocol analysis. The average age of the volunteers was around 55 years old, and the ratio of men to women was close to 1:1.8. The specific characteristics of the volunteers are shown in Table S2.

#### 3.5.2. Effect of *Lactobacillus* on *H. pylori* Load and Gastrointestinal Symptoms in Volunteers

**Figure 4:**
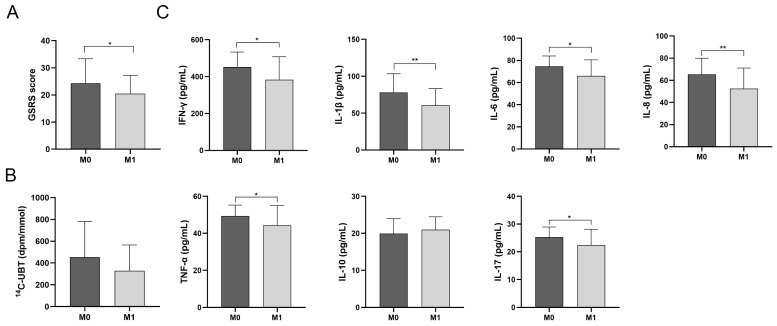
Probiotics modulate gastrointestinal response and inflammatory cytokine balance in patients with *H. pylori* infection. (**A**) Comparison of mean GSRS scores between the two groups before and after taking probiotics. (**B**) Comparison of mean ^14^C-UBT test values between the two groups before and after taking probiotics. (**C**) Comparison of inflammatory cytokines between the two groups before and after taking probiotics: IFN-γ, IL-1β, IL-6, IL-8, TNF-a, IL-10, IL-17 in serum. Error bars represent the standard deviation of biological repetition (*n* = 37). *, *p* < 0.05; **, *p* < 0.01.

The results of ^14^C-UBT can reflect the density of *H. pylori* in *H. pylori*-infected patients. As can be seen from the test results, there was a decreasing trend of ^14^C-UBT values after the volunteers took the probiotics (454.30 ± 327.00 vs. 328.35 ± 237.19, *p* = 0.06) (Figure 4B); 27 volunteers showed a significant decrease in the test value; 4 volunteers showed negative results with a test value below 100.

After 1 month, there was a significant difference in mean GSRS scores between the M0 and M1 groups (24.30 ± 9.05 vs. 20.49 ± 6.73, *p* < 0.05) (Figure 4A). Specifically, a comparison of gastrointestinal symptoms before and after taking probiotics showed that significantly fewer patients in the M1 group experienced throat discomfort (18% vs. 27%), halitosis (27% vs. 35%), chest pain (8% vs. 16%), acid regurgitation (13% vs. 21%), abdominal distension (32% vs. 59%), and diarrhea (21% vs. 43%) than in the M0 group (Table 3).

#### 3.5.3. Effect of *Lactobacillus* on Immunity-Related Indicators in Volunteers

As can be seen from the results, the pro-inflammatory factors TNF-α, IFN-γ, IL-1β, IL-6, IL-8, and IL-17 in the serum of the volunteers in the M1 group were significantly reduced compared to the M0 group (*p* < 0.05). The change of anti-inflammatory factor IL-10 in the serum was not very obvious (Figure 4C). Therefore, probiotics can play a role in regulating the immune level of *H. pylori*-infected patients, especially in regulating the level of pro-inflammatory factors.

#### 3.5.4. Gut Microbiota Analysis

**Figure 5:**
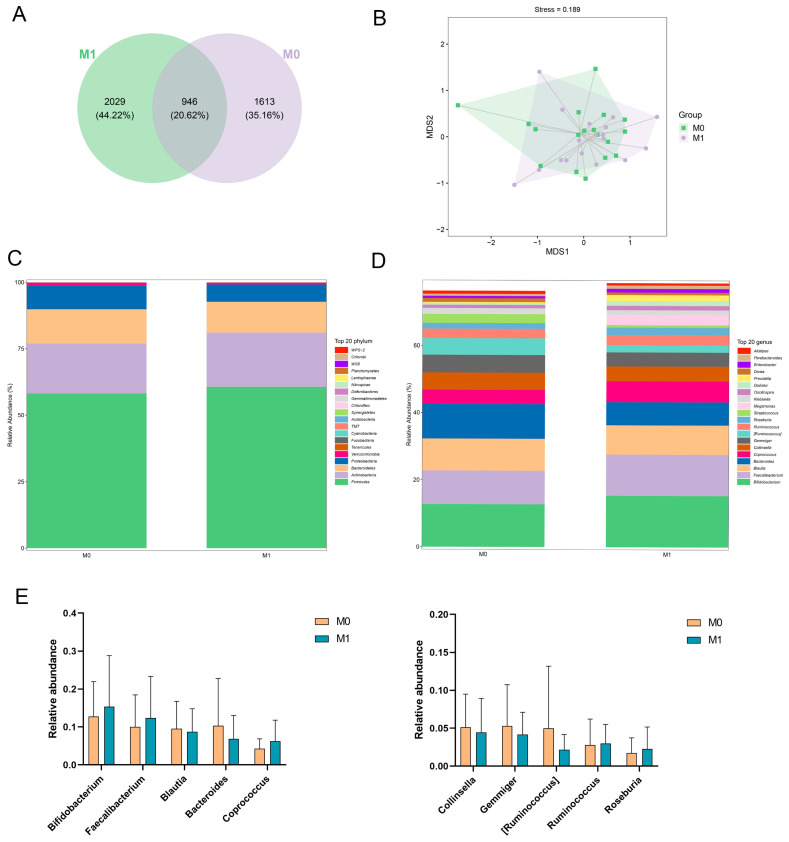
Effect of probiotics on microbial diversity and gut microbiota composition. (**A**) Venn plot that illustrated the observed ASV counts in samples. (**B**) NMDS analysis based on Bray–Curtis. (**C**) The gut microbiota composition at the phylum level. (**D**) The gut microbiota composition at genus level. (**E**) Relative abundance of the ten most predominant gut microbiota at genus level.

There was a trend towards increased alpha-diversity (as detected at the species level by Observed species, Chao-1 and Shannon’s index) in the gut microbiota of the M1 group compared to M0 (Table 4). The mutual and unique ASVs among each group were expressed by the Venn plot in Figure 5A. In total, 946 ASVs were mutual by two groups, with the M1 group having the largest count of specific ASVs (2029). Meanwhile, 1613 unique ASVs were observed in the M0 group. These results agreed with the richness index and diversity index in Table 4. Beta-diversity analyses were performed to assess differences in diversity between groups. Bray–Curtis-based NMDS analyses showed no significant segregation of gut microbial structure between groups, and changes were not significant (Figure 5B).

To identify differences in the gut microbiota between the two groups, we analyzed the top 20 most abundant microorganisms at the phylum level. It was found that the dominant phylum in both groups was Firmicutes and Actinobacteria (relative abundance >18%), and their relative abundance was slightly higher in the M1 group compared to the M0 group (Figure 5C). Meanwhile, the relative abundance of Proteobacteria and Verrucomicrobia was reduced in the M1 group compared to the M0 group. It has been shown that the intestinal microecology is disturbed when the host is infected with *H. pylori* and that the administration of probiotics improves gastrointestinal symptoms and alters the ratio of Firmicutes, among others [47].

When analyzing the community structure at the genus level in detail, it was found that the proportions of *Bifidobacterium* and *Faecalibacterium* were higher in the M1 group than in the M0 group (Figure 5D,E). Additionally, the proportion of *Bacteroides*, [*Ruminococcus*] was reduced in group M1 compared to group M0. Although there was a change in the community structure in the M1 group compared to the M0 group, there was no significant change, which may be caused by the large individual differences. Taking the dominant group *Bifidobacterium* as an example, it enables other bacterial populations to stably colonize the intestines, and this strong bacterial correlation shapes the establishment, stability, and evolution of the intestinal microbiota and maintains intestinal microecological health [48]. Thus, probiotics have a positive regulatory effect on the gut microbiota.

#### 3.5.5. Differential Metabolite Analysis and Related Metabolic Pathway Prediction

**Figure 6:**
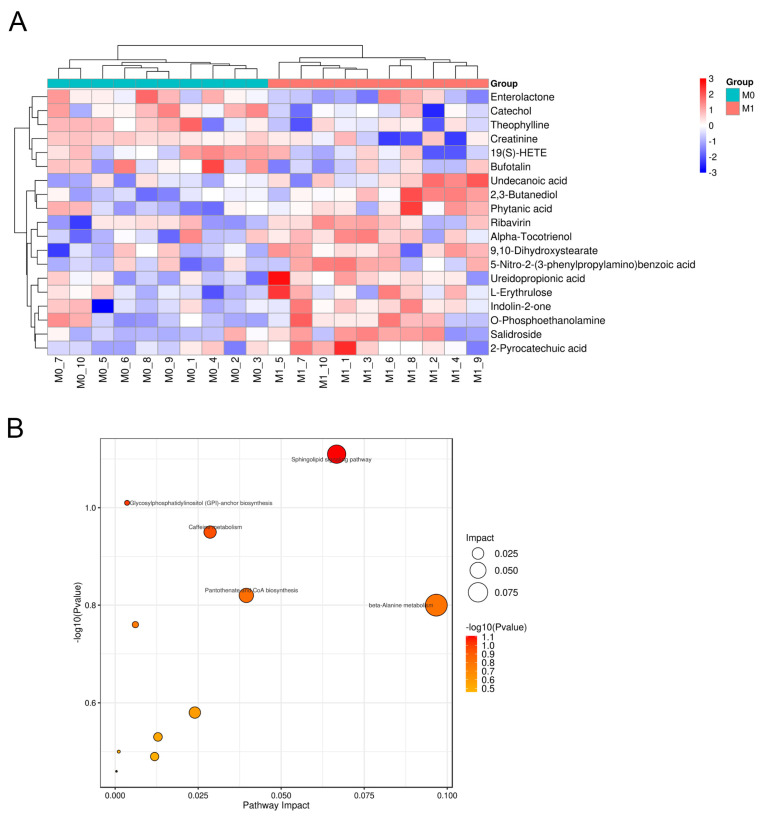
Effect of probiotic intervention on differential metabolites. (**A**) Clustering heat map of differential metabolites before and after the probiotic intervention. (**B**) Bubble diagram of metabolic pathway influencing factors.

Differential metabolite analysis and metabolic pathway prediction were performed to investigate the changes in relevant metabolites before and after the probiotic intervention. A total of 19 differential metabolites with significant changes were screened in MS secondary characterization mode (Figure 6A). The metabolic contents of Ureidopropionic acid and O-Phosphoethanolamine were significantly increased in the M1 group compared to the M0 group, and the increase in O-Phosphoethanolamine was particularly significant. The relative contents of other metabolites, such as Indolin-2-one, Undecanoic acid, Phytanic Acid and others showed a certain degree of increase. Significantly different metabolites were introduced into KEGG (Kyoto Encyclopedia of Genes and Genomes) for metabolic pathway analysis to identify the major signaling pathways and biometabolic pathways in which they are involved. The metabolic pathways predominantly involved with O-Phosphoethanolamine encompass the Sphingolipid signaling pathway, Glycosylphosphatidylinositol (GPI)-anchor biosynthesis, Sphingolipid metabolism, and Glycerophospholipid metabolism. The most influential pathway is the Sphingolipid signaling pathway (Figure 6B). An analysis of the KEGG metabolic pathways involved in differential metabolites before and after the intervention with probiotics—the Sphingolipid signaling pathway—is shown in Figure S3.

## 4. Discussion

The increase in *H. pylori* antibiotic resistance has led to a decline in the eradication rate of standard triple therapy [49]. Some studies suggest that probiotics are considered alternative or adjunctive therapeutic approaches to improve the eradication rate of *H. pylori* and reduce drug-related side effects [50,51].

In this study, firstly, three strains of *Lactobacillus plantarum*, Q21, Q25, and QA85, were screened by utilizing the probiotic properties of *Lactobacillus* as well as the inhibitory properties of *H. pylori* growth. Our results indicated that they have the potential to withstand the acidic environment of the stomach. Moreover, these strains exhibit strong hydrophobicity. Strains that are highly hydrophobic may have protein-like substances on their cell surfaces, which may have a better self-protective effect on the cells [52]. Zuo et al. reported that autoaggregation of probiotic strains may be one of the mechanisms to maintain the viability of the strains and increase the resistance of the human upper gastrointestinal tract to pathogenic bacteria. Autoaggregation allows probiotics to form spatial site barriers to impede the colonization of the gastrointestinal tract by pathogenic bacteria such as *H. pylori* [53]. Certainly, the aggregation of pathogenic bacteria by *Lactobacillus* can make it easier to expel pathogenic bacteria from the intestine [54]. All three strains screened in this study possessed strong autoaggregation and coaggregation abilities, enabling them to hinder the colonization of *H. pylori*.

Zheng et al. reported that lactobacilli have anti-*H. pylori* effects, which depend on their secreted components and lactic acid-mediated bactericidal activity [55,56,57]. Urease activity is an important factor in the colonization of *H. pylori*, altering the properties of mucus by neutralizing acidic pH [58]. Our results showed that Q21, Q25 and QA85 had high anti-H. pylori activity, which may also be related to their secretions.

Previous studies have found that probiotics can repair the mucosal permeability of the gastric mucosa, form a mucosal barrier, and inhibit the adhesion of pathogenic bacteria such as *H. pylori* [59]. Do et al. reported that *L. rhamnosus* JB3 has demonstrated the ability to inhibit the binding of *H. pylori* to cells [60]. Our research found that Q21, Q25, and QA85 exhibit strong colonization ability in gastric epithelial AGS cells. Additionally, they inhibit the adhesion of *H. pylori* to AGS cells, further confirming the ability of *Lactobacillus* to impede the colonization and adhesion of *H. pylori* on gastric mucosa. *Lactobacillus* achieve their anti-adhesive effects on *H. pylori* through the following mechanisms: competing for epithelial cell binding sites, releasing antimicrobial compounds to kill *H. pylori*, and reducing the expression of adhesin genes [61].

The manifestation of *H. pylori*-induced gastritis has been associated with the release of a wide variety of inflammatory mediators, such as chemokines and cytokines [62]. *H. pylori* can release CagA directly into host cells, leading to the production of IL-8 as well as other inflammatory factors by epithelial cells [63,64,65]. Studies have also demonstrated that probiotics inhibit the production of pro-inflammatory factors, such as IL-8, leading to a reduction in neutrophil infiltration in the gastric mucosa. Moreover, they promote the generation of anti-inflammatory factors like IL-10, eliciting an immune response in Th2 cells [66]. Therefore, probiotics can modulate the immune response of the host by adhering to epithelial cells, and in particular, they can reduce the inflammatory response of the stomach by modulating the balance of inflammatory factors [67]. In this study, we found that Q21, Q25 and QA85 were able to reduce the expression of pro-inflammatory factor genes, such as IL-8, with potential immunomodulatory activity. Probiotics are capable of generating a wide variety of immune responses due to strain specificity, and the immune status of the host affects the immune response [68]. Therefore, it is necessary to further investigate the mechanisms by which the strains regulate inflammatory signaling.

In vitro studies revealed that Q21, Q25, and QA85 have anti-*H. pylori* potential, and we further validated their effects through in vivo studies. In this own-control trial of volunteers taking probiotics, the ^14^C-UBT assay showed a decreasing trend in the load of *H. pylori* after one month of taking probiotics (454.30 ± 327.00 vs. 328.35 ± 237.19, *p* = 0.06). However, probiotics alone cannot eradicate *H. pylori*. Meanwhile, the use of probiotics can significantly improve the gastrointestinal symptoms of patients, especially for symptoms such as abdominal distension and diarrhea. Some studies reported that *Lactobacillus*, such as *L. acidophilus*, *L. casei* Shirota, and *L. johnsonii* La1, may reduce the bacterial load of *H. pylori* [69]. Chen et al. [70] observed that although probiotics were effective in reducing the bacterial load of *H. pylori*, the beneficial effect disappeared after a week of discontinuing probiotics. Therefore, the use of probiotics alone is not recommended for *H. pylori* eradication. However, probiotics in combination with triple or quadruple therapy may be a better option.

Some studies have shown that probiotics can reduce the severity of *H. pylori*-related illnesses [71,72]. Some in vitro studies have shown that *L. casei*, *L. acidophilus* and *B. lactis* strains have inhibitory and bactericidal activity against *H. pylori* [71]. Probiotics are thought to reduce gastritis in the sinuses and body of the stomach [72]. Probiotics not only inhibit *H. pylori*, but also alter the host’s immune response to reduce *H. pylori*-related gastric inflammation by inactivating the SMAD7 and NF-kB pathways [73]. Our findings also revealed that the levels of pro-inflammatory factor proteins such as TNF-α, IL-6, and IL-8 were suppressed in the patients, confirming that probiotics can modulate the immune balance of the host.

Probiotics have shown a role in preventing or reducing *H. pylori* infection-associated diarrhea. Emara et al. and Chotivitayatarakorn et al. found that probiotics significantly reduced the incidence of diarrhea in a 14-day triple therapy study in combination with probiotics [74,75]. In our study using only probiotics alone, we found that significantly fewer patients experienced diarrhea after taking probiotics than before taking probiotics (21% vs. 43%). This result suggests that probiotics have a positive moderating effect on patients’ discomfort reactions such as diarrhea. Hauser et al. found that a 14-day triple therapy combining *L. rhamnosus* GG and *Bifidobacterium* significantly reduced the frequency of abdominal distension [76]. Our conclusions were similar, but unlike their study, we studied the probiotic combination of *Lactobacillus plantarum* Q21, Q25, and QA85 and did not combine it with triple therapy.

Dysbiosis of the gut microbiota has been reported to be associated with an increased risk of a number of diseases [77]. Probiotics have been suggested to have the potential to treat these diseases by modulating the gut microbiota [78]. However, there are still no clear conclusions as to whether or not probiotics alter the diversity or composition of the gut microbiota [33]. Most studies have shown that probiotics did not significantly affect the diversity of the gut microbiota [79,80]. Rahayu et al. found that giving volunteers *Lactobacillus plantarum* Dad-13 powder for 90 days resulted in a significant increase in alpha diversity and bacterial abundance of the gut microbiota [81]. It has also been found that taking *Lactobacillus paracasei* for 4 weeks can change the composition of the fecal microbiota [82]. Our study found that alpha diversity changed but not significantly after taking probiotics, and beta diversity was similar, and that individual differences may be an important factor affecting diversity. It was also found that there was no significant change in relative abundance at the genus level before and after taking probiotics. Our results are consistent with the results of several studies using only probiotics. For example, Chen et al. [70] found that although the use of a combination of *Lactobacillus acidophilus* and *Lactobacillus rhamnosus* for 4 weeks reduced the bacterial load of *H. pylori*, no significant changes in the diversity and composition of the intestinal microbiota, although fluctuations in the diversity and composition of the intestinal microbiota, were observed. Different results may be due to different treatment regimens, average age of patients, probiotic types, dosages, and length of use cycles.

Probiotics cause certain effects on fecal metabolites. These changes in metabolites have a great degree of correlation with various physiological and pathological processes of the organism. We screened the fecal differential metabolites of volunteers before and after taking probiotics and found that there were 19 differential metabolites with significant changes, especially the content of O-Phosphoethanolamine increased significantly after probiotic intervention. A search for its associated metabolic pathways revealed that the main metabolic pathway involved is the Sphingolipid signaling pathway. O-phosphoethanolamine is a phospholipid molecule. This compound plays an important role in living organisms, especially in the construction of cell membranes and metabolic pathways [83,84,85]. The Sphingolipid signaling pathway has a potentially important role in regulating cellular physiological processes, maintaining tissue homeostasis, and in the prevention and treatment of disease [85]. The study of this metabolic pathway has important implications for disease development and the search for therapeutic approaches.

This study has some strengths. Firstly, we recruited volunteers who were allowed to take probiotics as part of their daily routine, which assessed the effectiveness of probiotics in people’s normal life diets that do not exclude outside influences. Secondly, most of the volunteers in this study were around 55 years old, making it more applicable to an older population. Furthermore, probiotics are natural and harmless microbial agents that do not pose drug-like hazards when used on a daily basis. Probiotics can also be supplemented in routine treatment plans for *H. pylori*. However, there are some limitations to this study. This study was a self-controlled trial before and after taking probiotics and had a small sample size. In addition, we only recorded the bacterial load within 1 month of taking the probiotic, so we cannot know whether the inhibitory effect of the probiotic will persist beyond 1 month. Finally, fecal samples were not collected at additional time points, thus preventing comprehensive monitoring of the dynamics of the gut microbiota.

## 5. Conclusions

In this study, we screened candidate probiotic strains *Lactobacillus plantarum* Q21, Q25, and QA85 with strong anti-*H. pylori* ability and investigated their efficacy in alleviating symptoms associated with *H. pylori* infection. These strains, given to volunteers in the form of a probiotic powder, have been shown to result in a downward trend in the bacterial load of *H. pylori*, lower GSRS scores, and improve the severity and frequency of uncomfortable reactions such as abdominal distension and diarrhea, and reduce the inflammatory response. However, their role in regulating the structure and function of the gut microbiota was not significant, while their effect on fecal metabolites was more pronounced. The use of probiotics appears to have emerged as a low-cost and safer adjunctive therapy compared with standard triple or quadruple therapy, which may be one of the ideal strategies for alleviating *H. pylori* infection.

## Figures and Tables

**Table 1:** Specific primers used for RT-qPCR.

Target Gene	Primer Sequence (5′ to 3′)	Tm	Size of Amplicon (bp)
TNF-α	F:TTTGATCCCTGACATCTGGA	55.83	112
	R:GGCCTAAGGTCCACTTGTGT	59.60	
IL-6	F:GACAGCCACTCACCTCTTCA	59.32	457
	R:CGCAGAATGAGATGAGTTGT	55.87	
IL-8	F:ACTGAGAGTGATTGAGAGTGGAC	59.49	112
	R:AACCCTCTGCACCCAGTTTTC	60.75	
IL-10	F:AGGGAGGATGAGTGATTTGC	57.27	783
	R:AACTGGGAGGAACACTGACC	59.23	
β-actin	F:GACCTCTATGCCAACACAGT	57.23	139
	R:AGTACTTGCGCTCAGGAGGA	60.61	

IL: interleukin; F: forward; R: reverse.

**Table 2:** Results of the sensitivity of *Lactobacillus* to 22 antibiotics.

Class	Antibiotic	Content	Sensitivity			
			LGG	Q21	Q25	QA85
Penicillins	Penicillin	10 U	R	R	R	S
	Oxacillin	1 μg	R	R	R	S
	Ampicillin	10 μg	S	S	S	S
Aminoglycoside	Gentamicin	10 μg	I	R	R	R
	Streptomycin	10 μg	R	R	R	R
	Kanamycin	30 μg	R	R	R	R
Tetracyclines	Tetracycline	30 μg	S	I	S	I
	Doxycycline	30 μg	S	S	S	S
Cephalosporins	Imipenem	10 μg	S	S	S	S
	Ceftazidime	30 μg	S	R	R	R
	Cefotaxime	30 μg	I	R	R	R
	Cefuroxime	30 μg	S	S	I	S
Macrolide	Erythromycin	15 μg	I	I	I	I
Quinolones	Ciprofloxacin	5 μg	R	R	R	R
	Norfloxacin	10 μg	R	R	R	R
	Levofloxacin	5 μg	R	R	R	R
Folate metabolism pathway inhibitors	Sulfafurazole	300 μg	R	R	R	R
	Trimethoprim-sulfamethoxazole	23.75/1.25 μg	S	S	S	S
Glycopeptide	Vancomycin	30 μg	R	R	R	R
Chloramphenicol	Chloramphenicol	30 μg	S	S	S	S
Rifamycins	Rifampicin	5 μg	S	R	I	I

R: resistant; I: intermediate; S: susceptible.

**Table 3:** Comparison of symptoms in volunteers before and after taking probiotics.

Symptoms	Before (M0)	After (M1)
	*n* (%)	*n* (%)
Epigastric pain	13 (13.5)	13 (13.5)
Chest pain	16 (16.2)	8 (8.1)
Acid regurgitation	21 (21.6)	13 (13.5)
Nausea	10 (10.8)	10 (10.8)
Bowel sound	21 (21.6)	18 (18.9)
Abdominal distension	59 (59.5)	32 (32.4)
Throat discomfort	27 (27.0)	18 (18.9)
Halitosis	35 (35.1)	27 (27.0)
Constipation	37 (37.8)	35 (35.1)
Diarrhea	43 (43.2)	21 (21.6)

Note: Values provided as *n* (%).

**Table 4:** The gut microbial diversity indices.

Group	Richness Index		Diversity Index	
	Observed Species	Chao1	Simpson	Shannon
M0	262.69 ± 80.34	275.99 ± 82.32	0.93 ± 0.04	5.12 ± 0.74
M1	291.82 ± 119.62	305.04 ± 120.59	0.93 ± 0.05	5.31 ± 0.87

## Data Availability

The original contributions presented in the study are included in the article/Supplementary Materials, further inquiries can be directed to the corresponding author.

## Supplementary Materials

The following supporting information can be downloaded at: https://www.mdpi.com/article/10.3390/foods13121851/s1, Figure S1: Studies on the properties of *Lactobacillus*; Figure S2: Typical diagram of *Lactobacillus* antibiotic susceptibility; Figure S3: Prediction of the metabolic pathway before and after intervention with probiotics; Table S1: Strains conservation information; Table S2: Demographic characteristics of volunteers.
