# Supplementary material for: Screening Probiotics for Anti-Helicobacter pylori and Investigating the Effect of Probiotics on Patients with Helicobacter pylori Infection
Source: Foods. 2024 Jun 13;13(12):1851. doi: 10.3390/foods13121851 (PMC11202727; doi:10.3390/foods13121851)
Supplement: Supplementary file 1 [file foods-13-01851-s001.zip › foods-3006332-supplementary.pdf]

Table S1. Strains conservation information.

Strains	Collection Address	Depository Number
Q21	Guangdong Microbial Culture Collection Center (GDMCC)	GDMCC No. 63277
Q25	Guangdong Microbial Culture Collection Center (GDMCC)	GDMCC No. 63278
QA85	Guangdong Microbial Culture Collection Center (GDMCC)	GDMCC No. 61192
TSL-6	Guangdong Microbial Culture Collection Center (GDMCC)	GDMCC No. 61242
ZCJ	Guangdong Microbial Culture Collection Center (GDMCC)	GDMCC No. 61402
QX(A)-4	Guangdong Microbial Culture Collection Center (GDMCC)	GDMCC No. 61617
GL-5	China General Microbiological Culture Collection Center (CGMCC)	CGMCC No. 18988

Table S2. Demographic characteristics of volunteers.

Characteristics	Volunteers（N=37）	
Men/Women	13/24	
Smoking	10/37	
Drinking	17/37	
	**Mean**	**SD**
Age	55.70	12.90
Height (cm)	163.89	6.72
Weight (kg)	63.62	8.45

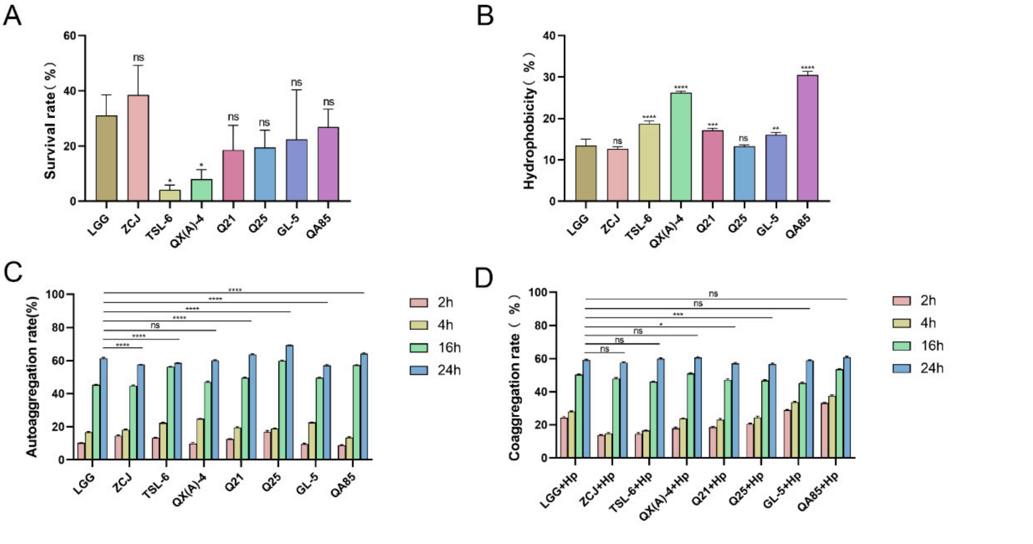

Figure S1. Studies on the properties of *Lactobacillus*. (A) Survival rate of *Lactobacillus* in simulated gastric juice at pH 3.0. (B) Hydrophobic properties of *Lactobacillus*. (C) *Lactobacillus* autoaggregation properties. (D) Coaggregation properties of *Lactobacillus* with H. pylori. Error bars represent the standard deviation of biological triplicates. *, $p < 0.05$; **, $p < 0.01$; ***, $p < 0.001$; ****, $p < 0.0001$; ns, no significant difference.

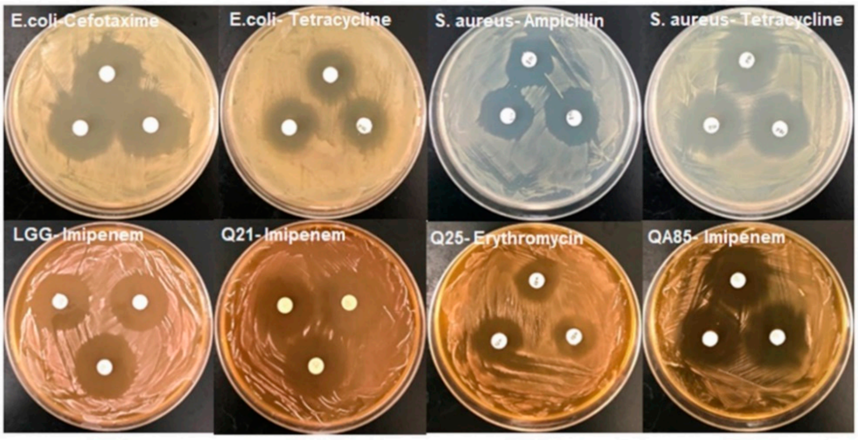

Figure S2. Typical diagram of *Lactobacillus* antibiotic susceptibility. E. coli and S. aureus were used as the control bacteria.

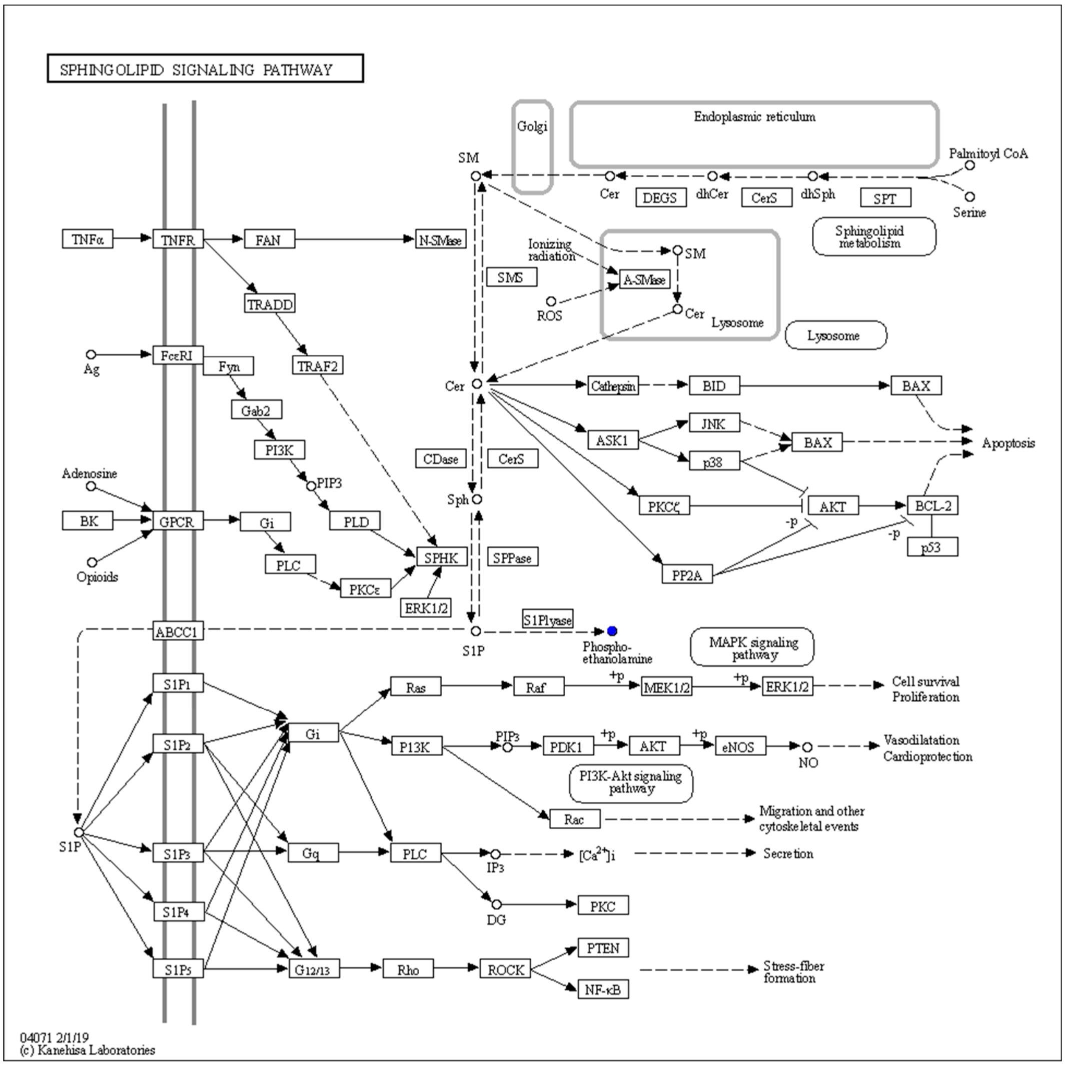

Figure S3. Prediction of metabolic pathway before and after intervention with probiotics. The KEGG metabolic pathway analysis diagram–Sphingolipid signaling pathway.
